# The role of macrophage subtypes and exosomes in immunomodulation

**DOI:** 10.1186/s11658-022-00384-y

**Published:** 2022-10-03

**Authors:** Abdulwahab Teflischi Gharavi, Niloofar Asadi Hanjani, Elaheh Movahed, Mohammad Doroudian

**Affiliations:** 1 Department of Cell and Molecular Sciences, Faculty of Biological Sciences, Kharazmi University, Tehran, 14911-15719 Iran; 2 Wadsworth Center, New York State Department of Health, Albany, New Year USA

**Keywords:** Exosomes, Inflammation, Macrophages, Macrophage polarization

## Abstract

Macrophages are influential members of the innate immune system that can be reversibly polarized by different microenvironment signals. Cell polarization leads to a wide range of features, involving the migration, development, and organization of the cells. There is mounting evidence that macrophage polarization plays a key role in the initiation and development of a wide range of diseases. This study aims to give an overview of macrophage polarization, their different subtypes, and the importance of alternatively activated M2 macrophage and classically activated M1 macrophage in immune responses and pathological conditions. This review provides insight on the role of exosomes in M1/M2-like macrophage polarization and their potential as a promising therapeutic candidate.

## Introduction

Macrophages and their phagocytosis activity were first discovered by Elie Metchnikoff and Paul Ehrlich in 1908 [1–3]. The mononuclear phagocyte system (MPS) is a professional phagocyte that comprises dendritic cells (DCs), blood monocytes, and tissue macrophages [4]. Macrophages are mononuclear cells that are the most plenteous and widespread immune cells and are involved in phagocytosis, homeostasis, and remodeling after injury and are necessary during organ development [5–7]. Macrophages can originate from local tissue-resident macrophages with a self-renewed ability or blood monocytes. Bone marrow progenitor-derived monocytes migrate to tissue by receiving stimuli signals and then become macrophages [4, 8]. Macrophages have plastic characteristics. These characteristics enable them to switch their phenotypes and functions in connection with various microenvironmental signals [9].

Macrophages have been observed in many tissues. They are categorized on the basis of their location and function, for instance, microglial cells in the central nervous system (CNS) with the ability to clear defective neurons; alveolar macrophages in the lung, which are needed for lung homeostasis; osteoclasts in bone with bone remodeling activity [10–12]; and Kupffer cells, which are the most lavish macrophages present in liver [13–15]. Macrophages are innate immune cells that can affect a variety of processes, including tissue repair, angiogenesis, and immunomodulation [16, 17]. Macrophages gain different phenotypes and functions under normal condition or during disease. Macrophages’ ability to change their functions in response to different signals is known as polarization, which is a multifactorial process [18]. This is a key mediator of different diseases, including autoimmune diseases [19], glycolipid metabolic disorders [20], neurology disorders [21], cardiovascular diseases [3], and cancers [22]. Different polarized macrophages, M1 (classically activated macrophages) and M2 (alternatively activated macrophages), express diverse cell surface markers and factors (Table 1) [23].

**Table 1:** Macrophage subtypes and their characteristic markers and stimuli

Macrophage types	Suggested roles	Markers	Different stimulator factors	References
M1	Pro-inflammation, microbicidal effect, tumor resistance	IL-6, IL-10 (low), IL-12 (high), iNOS, CD80, CD86, CXCL9, CXCL10, CXCL11, CCL15, CCL20, CCL22, TLR2, TLR4, MHCII, TNF-α	IL-1, IL-6, IL-12, CXCL1-3, CXCL-5, CXCL8-10, CCL2, Type I IFN, IFN-γ, TNF-α, STAT1, iNOS, LPS, M-CSF, NF-κB, IRF5 miR-155, miR-125b, DNMT1, DNMT3b, HDAC3	[19, 28–31]
M2a	Allergy, profibrotic, anti-inflammatory, wound healing	IL-10, IL-1R, IL-27Rα, CCL1, CCL17, CCL18, CCL22, CD11b, CD45, CD206, YM1, RELMα, IGF1, DCIR, Stabilin 1, Factor XIII-A, Ly6C, TREM-2, DC-SIGN	IL-4, IL-10, IL-13, (PPAR-γ)	[19, 28, 32–35]
M2b	Th2 activation, immune regulation, promoting infection, tumor progression	IL-6, TNF-α, CD86, SPHK1	IL-1β, LPS	[28, 31, 36]
M2c	Immunosuppression, phagocytosis, tissue repair, matrix remodeling	IL-10, CXCL13, CD163, CD206, CXCR4, TGF-β, MerTK,	IL-10, glucocorticoids IL-6, IL-10, TNF-α, TLR,	[28, 36, 37]
M2d	Tumor progression, angiogenesis, clearance of apoptotic tissue	IL-10, VEGF, TGF-β,	LPS	[28, 36, 38]

CCL, C–C chemokine ligand; CXCL, C–X–C chemokine ligand; CXCR, C–X–C chemokine receptor; DCIR, dendritic cell immunoreceptor; IFN, interferon; IFNγ, interferon-γ; IL, interleukin; IL-1R, IL-1 receptor; IL-27Rα, IL-27 receptor α-chain; iNOS, inducible nitric-oxide synthase; RELMα, resistin-like molecule-α; SPHK1, sphingosine kinase 1; TLR, Toll-like receptor; CD, cluster of differentiation; TGF-β, transforming growth factor-β; STATs, signal transducer and activator of transcription; PPAR-γ, peroxisome proliferator-activated receptor gamma; TNF-α, tumor necrotic factor-α; YM1, chitinase-like protein 3; LPS, lipopolysaccharides; VEGF, vascular endothelial growth factor; ICAM, intercellular adhesion molecule; DC-SIGN, dendritic cell-specific ICAM-grabbing non integrin; IRF5, interferon regulatory factor 5; HDAC3, histone deacetylase 3; MerTK, myeloid epithelial reproductive tyrosine kinase; M-CSF, macrophage-colony-stimulating factor; DNMT, DNA methyl transferase; miRs, microRNAs; NF-κB, nuclear factor-κB; MHCII, major histocompatibility complex II; IGF, insulin-like growth factor; Ly6C, lymphocyte antigen 6 complex; TREM-2, triggering receptor expressed on myeloid cells 2

**Fig. 1:**
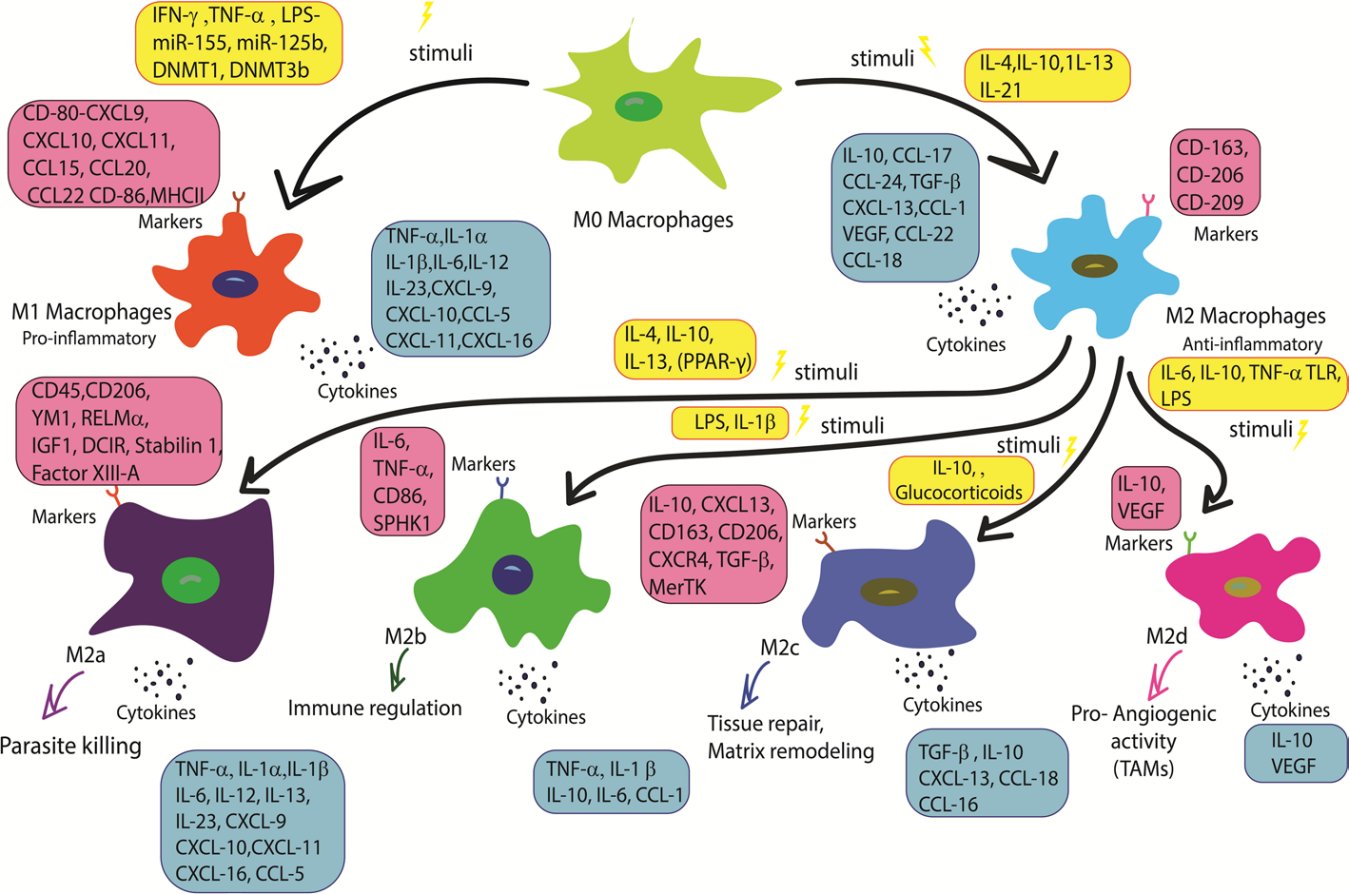
Macrophage polarization phenotypes and subtypes according to their different characteristics have many roles in immune responses. M1 macrophages have a pro-inflammatory role by their cytokines, but M2 macrophages, which are divided into four subtypes, have many different roles. For example, M2a macrophages play an important role in parasite killing, M2b macrophages function as immune system regulators, M2c macrophages assist in the wound healing process, and M2d macrophages have a pro-angiogenic role and are very important in tumor progression. CCL, C–C chemokine ligand; CXCL, C–X–C chemokine ligand; CXCR, C–X–C chemokine receptor; DCIR, dendritic cell immunoreceptor; IFN, interferon; IFNγ, interferon-γ; IL, interleukin; RELMα, resistin-like molecule-α; SPHK1, sphingosine kinase 1; TLR, Toll-like receptor; CD, cluster of differentiation; TGF-β, transforming growth factor-β; PPAR-γ, peroxisome proliferator-activated receptor gamma; TNF-α, tumor necrotic factor-α; YM1, chitinase-like protein 3; LPS, lipopolysaccharides; VEGF, vascular endothelial growth factor; MerTK, myeloid epithelial reproductive tyrosine kinase; DNMT, DNA methyl transferase; miRs, microRNAs; MHCII, major histocompatibility complex II; IGF, insulin-like growth factor

The polarization of macrophages is a dynamic process, so it can be reversed by different microenvironment conditions in different biological conditions and diseases. Macrophages can polarize with more than two forms [24]. M2 macrophage, also called activated or healing macrophage, was first described in the 1990s [25]. Subsets of M2 macrophages include M2a, M2b, M2c, and M2d, with different properties such as cell markers, cytokines, and functions, i.e., M2a enhances cell growth and tissue repair while M2b, M2c, and M2d play crucial roles in inflammation, phagocytosis, and tumor progression, respectively [16, 26]. M2d macrophages are the main element of tumor microenvironment and are generally called tumor-associated macrophage (TAM). They could promote cancer-related processes such as progression and invasion of cancerous cells (Fig. 1) [27, 28]. There is a dynamic balance between different types of macrophages that could cause a variety of diseases when it is disturbed. The number of tissue-resident macrophages is regulated by colony-stimulating factor-1 (CSF-1) or macrophage-colony-stimulating factor (M-CSF), interleukin-34 (IL-34), and colony-stimulating factor-1 receptor (CSF-1R) [4]. M1 macrophages are the first agents of protection in blocking the intracellular pathogens, and their activation can promote T-helper lymphocytes type 1 (Th1) polarization. M1 macrophages release great amounts of some cytokines that have pro-inflammatory roles, including tumor necrotic factor-α (TNF-α), monocyte chemo attractant protein-1 (MCP-1), IL-6, IL-1, IL-12, type 1 interferons (IFNs), inducible nitric oxide synthase (iNOS), and C–X–C motif chemokine ligands (CXCLs) such as CXCL1-3, CXCL5, and CXCL8-10.

**Fig. 2:**
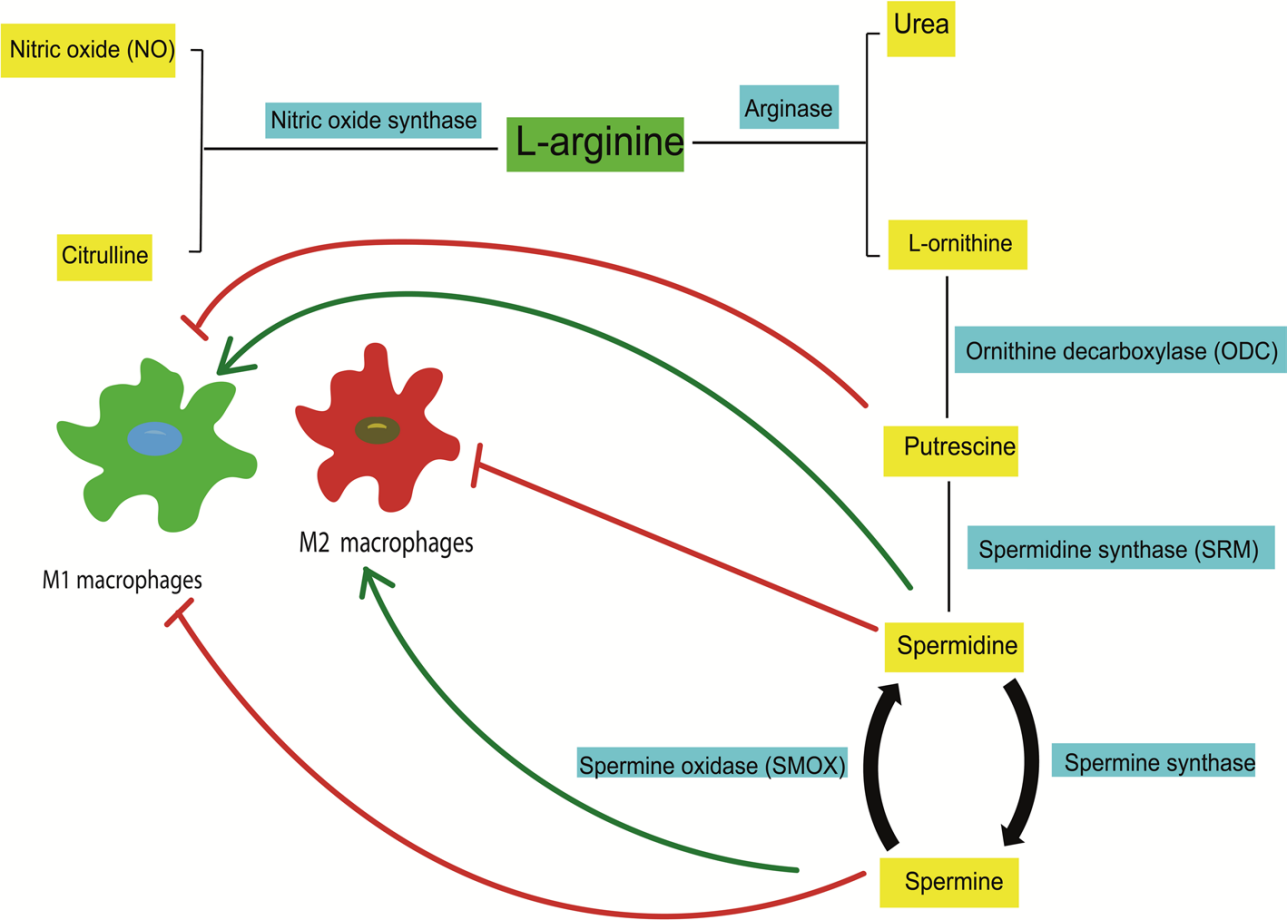
Mammalian arginine metabolism pathways and M1 and M2 macrophage polarization. Arginine metabolism can be derived via NOS or arginase. NOS, which is expressed in M1 macrophages, causes arginine metabolism to release NO and citrulline, and M2 macrophages synthesize arginase, which causes arginine metabolism to release ornithine and urea. Downstream pathways of ornithine include putrescine, spermidine, and spermine, which hydrolyze products of ODC, SRM, and spermine synthase, respectively. Spermine can be resynthesized to spermidine by SMOX. As shown in the figure, putrescine and spermine downregulate the polarization of the M1 macrophages. Spermidine upregulates M1 macrophage polarization. In the case of M2 macrophage polarization, spermidine and spermine have inhibitor and enhancer effects, respectively. M1 macrophages increase NOS, but M2 macrophages upregulate arginase and ODC. Both arginine metabolic pathways arrest each other. Enzymes are shown by blue boxes and metabolites by yellow boxes, respectively. NOS, nitric oxide synthase; NO, nitric oxide; ODC, ornithine decarboxylase; SRM, spermidine synthase; SMOX, spermine oxidase

M2 macrophages are involved in infections caused by fungal, parasitic, or helminthic pathogens and conversely express high level of dectin-1, DC-SIGN (CD209), mannose receptor (CD206), CD163, scavenger receptor A and B-1, C–C chemokine receptor 2 (CCR2), C–X–C motif chemokine receptor 1 (CXCR1), and CXCR2. M2 macrophages produce materials that play a role in tissue remodeling and repair, such as IL-10, chitinase-like protein 3 (YM1), macrophage and granulocyte inducer-form 1 (MgI1), and arginase-1 [4, 16]. Arginine metabolism pathways play a central role in macrophage polarization (Fig. 2) [29, 30]. Macrophage polarization is governed by the surrounding microenvironment, including cytokines and other components such as oligosaccharides, or by exosomes [22, 31]. On the other hand, epigenetic mechanisms such as chromatin remodeling, DNA methylation (DNAm), and histone modifications can control this process in connection with different factors. It was demonstrated that different levels of DNA methyl transferase 1 (DNMT 1), 3a and b, are expressed in M1 and M2 macrophages [4].

## The importance of macrophage polarization balance

Macrophage activation is necessary for the appropriate response against pathogen spreading in infected tissue. The process begins with pathogen-associated molecules releasing active danger signals that induce most tissue-resident macrophages to M1 polarization with nitrogen/oxygen-reactive agents and pro-inflammatory cytokine production ability. The next step is clearing cellular debris, then wound-healing signals commence M2 polarization with anti-inflammatory activity [26, 32–34]. Data validate the association between various diseases and the balance of M1/M2 macrophage polarization [35]. M1/M2 ratio and the regulation of macrophage polarization are very important. This ratio can signal progression of many inflammation-related diseases, for example, in psoriasis, which is a chronic inflammatory skin disorder. M1 macrophage markers are more abundant than M2 macrophage markers in psoriatic tissues [36]. Some pathogens and even tumors are able to reduce the M1/M2 ratio to avoid inflammation response [37–39]. For instance, some *Lactobacillus* are able to inhibit the formation of foam cells, which are a type of macrophage with lipoprotein ingestion activity [40]. Macrophage polarization is linked with some clinical conditions, including diabetes and obesity [3, 41], rheumatoid arthritis (RA) [42–44], chronic obstructive pulmonary disease [45], atherosclerosis [46, 47], non-alcoholic fatty liver disease (NAFLD) [48], osteoclastogenesis [49, 50], asthma [51], hypertension, and cardiovascular disease [52].

Balanced M1/M2 ratios are necessary for appropriate inflammatory response [16, 53]. Different stimuli factors or pathways involved in macrophage polarization can be promising candidates for therapeutic targets (Table 2) [4, 8, 54]. For instance, thiazolidinediones (TZDs), which target a member of the M2-like macrophage pathway, peroxisome proliferator-activated receptor gamma (PPAR-*γ*), are used for patients with type 2 diabetes (T2D) [55]. In a clinical study, it was observed that reducing the ratio of M1/M2 macrophages by blocking T-cell death-associated gene 8 (TDAG8), which has pro-inflammatory role, can attenuate RA progression (NSC745885) [56].

**Table 2:** Immunomodulatory drugs that target macrophage polarization

immunomodulatory drugs	Functions	References
Thapsigargin	Promote M2 polarization	[95]
Glucocorticoids	Trigger M2 polarization	[96]
Azithromycin	Promotes polarization from M1 to M2	[97]
5-Aminosalicylates (5-ASAs)	Inhibitory role in macrophage activation and inflammation suppressor	[98]
[6-(1-Methyl-4-nitroimidazol-5-yl)thiopurine]	Repressed nitric oxide synthase (iNOS) expression	[99]
Imiquimod	Restore pro-inflammatory of TAMs	[100]
PLX3397, PLX108-01 (pexidartinib)	Deplete macrophages	[101–103]
Trabectedin	Deplete macrophages	[104]
CP-690,550 (tofacitinib)	Inhibit modulate gene expression in macrophages	[105]
Hyaluronic acid oligosaccharides	Modulate macrophage polarity	[106]
Tocilizumab	M1 macrophage suppression	[107, 108]

Macrophages possess phagocytosis activity, which makes them able to capture nanoscale particles, and thus are appropriate candidates for targeting macrophages [57–61]. In a study, a bioactive nanodevice was designed to convert the M1 phenotype to the M2 phenotype. A nanodevice is a peptide-coated gold nanoparticle (GNP) that promotes inflammation resolution. It also can be used as a novel therapeutic agent for patients with acute lung injury (ALI) and acute respiratory distress syndrome (ARDS) [62]. It has been shown that nanoparticles, including designed miRNAs or siRNAs, switch macrophage polarization in some disease conditions [63, 64]. Clinical and experimental data have shown that M2 macrophages, which are TAMs, improve the growth, migration, invasion, and immunosuppressive activities of tumor cells [65–67]. Various therapeutic strategies targeting TAMs, depletion of the M2 macrophage ratio, or converting M2 macrophages to M1 macrophages are highlighted as potential strategies for suppressing tumor progression [65, 68–70]. TAMs can be used as novel targets in cancer therapy. For instance, a saponin component isolated from *Astragali radix*, called astragaloside IV (AS-IV), is reported to reduce tumor growth and metastasis by arresting the polarization of M2 macrophages through the adenosine monophosphate-activated protein kinase (AMPK) signaling pathway [65, 71].

Another study has demonstrated that M1 macrophage induction can increase cellular apoptosis and decrease tumor metastasis and chemotherapy resistance in mice with hepatocellular carcinoma (HCC) [72]. Macrophage targeting strategies in combination with chemotherapies exhibit more antitumor activity [73]. Among the autoimmune diseases caused by imbalanced M1/M2 ratio are systemic lupus erythematosus (SLE) [74], inflammatory bowel diseases (IBD) [75], autoimmune myocarditis [76], and autoimmune neuritis [77, 78]. Chronic inflammation is associated with age-related diseases such as cardiovascular disease, diabetes, and Alzheimer’s disease [5]. Much research has revealed that macrophages participate in the process of pregnancy [79] and could have important effects on preeclampsia, miscarriage, and preterm birth [32]. Different phenotypes and functions of macrophages are essential for each phase of pregnancy to establish and maintain pregnancy. Decidual macrophages participate in implantation, spiral artery remodeling, and angiogenesis of embryo, and they also protect the embryo from pathogens and maternal immune responses [80].

According to various studies, different polarized states of macrophages have been associated with diseases, including cystic fibrosis (CF) and asthma; high level of M2-polarized macrophages is correlated with higher-severity asthma [81]. Accumulating data have shown indispensable roles for M2b macrophages in cardiovascular diseases [82]. In addition to M1 and M2 macrophages, a “chimeric” M1–M2 type with mixed biological function and phenotype that can cause impaired inflammation conditions is described in some cases such as rheumatoid arthritis [83]. In fact, recent evidence shows a continuum of different macrophages with different markers and phenotypes that strongly depend on their microenvironment. Therefore, they can be considered as a spectrum [84–86], although simplified macrophage classification (M1/M2) is used for better understanding. M1 and M2 macrophages can be reprogrammed by different stimuli signals. These reversible changes are essential during inflammation and its resolution phases [87, 88]. Of note, the tumor microenvironment (TME) has a vital effect on cancer progression [89, 90]. Macrophages are able to alter their features according to the TME. Therefore, macrophage polarization can be considered as a therapeutic strategy for cancer treatment [66].

## Inflammation and the role of macrophage polarization in this process

Inflammation is a physiological condition that occurs in response to various situations, such as injury and infection. Acute inflammation is the first mechanism in responding to these conditions [91]. Uncontrolled acute inflammation may lead to chronic inflammation, which has been related to many diseases [92]. Sterile inflammation (SI) occurs by nonmicrobial factors such as chemical, physical, or metabolic stimuli, while nonsterile inflammation occurs by infection [93, 94]. Regenerative inflammation takes place in cases of low-grade damage or in highly regenerative tissues, such as the liver, and this type of inflammation plays a critical role in regeneration and repair [95]. Fibrotic inflammation occurs as a response to extensive damage or in poorly regenerative tissues, such as the myocardium. Macrophages play a crucial role in fibrotic inflammation [96]. Inflammation can lead to different immune responses via releasing molecular mediators. These mediators have roles in the direction of vascular responses, immune cell recruitment, macrophage polarization, pathogen clearance, repair of damaged tissue, and restoration of homeostasis. The balance of signal transducer and activator of transcription (STATs) activation has a very important role in macrophage polarization. The activation of STAT1 can lead to M1 macrophage polarization. This is important in the process of cytotoxicity and pro-inflammatory functions. On the contrary, some cytokines such as IL-4/IL-13 and IL-10 and activation of STAT3 and STAT6 can increase M2 polarization. Some other factors such as DNA methylation, chromatin remodeling, histone modification, and meta-inflammation, caused by chronic overnutrition and obesity, are involved in macrophage polarization. There are many clinical trials on macrophage polarization and inflammation (Table 3) [16, 97, 98]. Cardiac macrophages (CMs) are tissue-resident macrophages that are critical agents in the generation and development of cardiac inflammation, tissue remodeling, and repair. CMs are activated by the recognition of DAMPs or PAMPs. via cytokines released from inflammatory cells in the myocardium. An example of this is a promotion of M2 phenotype in dead cell clearance processes and an increase in the level of IL-10 and transforming growth factor-β (TGF-β), but pro-inflammatory cytokines such as TNF-α promote the M1 phenotype [99]. Unregulated immune responses mediated by macrophages may lead to chronic kidney disease (CKD). The balance of macrophage polarization between M1 phenotype and M2 phenotype is important in tissue injury.

**Table 3:** Clinical trials on macrophage polarization and inflammation

Status	Study title	Intervention	ClinicalTrials.gov identifier
Recruiting	Treatment of macrophage activation syndrome (MAS) with anakinra	•Drug: kineret •Drug: placebo	NCT02780583
Completed	Effect of liraglutide (Victoza) on inflammation in human adipose tissue and blood	•Drug: Victoza (liraglutide) with dietician monitoring •Other: placebo with dietician monitoring	NCT02650206
Completed	The effect of gut sterilization on macrophage activation in patients with alcoholic hepatitis	•Drug: combined vancomycin and gentamycin and meropenem	NCT03157388
Completed	Macrophage activation markers during sofosbuvir-based treatment regimens of chronic hepatitis C	•Drug: galactose •Procedure: gastroscopy •Procedure: liver biopsy •Procedure: FibroScan •Procedure: liver vein catheterization •Drug: sofosbuvir	NCT02528461
Unknown status	New candidate criteria for diagnosis of macrophage activation syndrome	–	NCT01095146
Completed	Exploration of immunity in Gaucher disease	–	NCT01358188
Completed	A study to investigate the safety and efficacy of emapalumab, an anti-IFN-gamma mAb in patients with systemic juvenile idiopathic arthritis (sJIA) or adult-onset Still’s disease (AOSD) developing macrophage activation syndrome/secondary HLH (MAS/sHLH)	•Drug: emapalumab	NCT03311854
Recruiting	sCD163 in patients with PBC—assessment of disease severity and prognosis	•Other: blood samples, FibroScan, and questionnaires	NCT02924701
Completed	A role for RAGE/TXNIP/inflammasome axis in alveolar macrophage activation during ARDS (RIAMA): a proof-of concept clinical study	•Other: RAGE/TXNIP/inflammasome axis	NCT02545621
Recruiting	sCD163 in patients with PBC—assessment of treatment response	•Other: blood samples •Device: FibroScan •Other: questionnaires •Biological: liver biopsy	NCT02931513
Completed	Downmodulating monocyte activation for HIV-1-associated neurocognitive disorders (HAND)	•Drug: atorvastatin (Lipitor) •Drug: placebo	NCT01600170
Completed	A trial of validation and restoration of immune dysfunction in severe infections and sepsis	•Drug: anakinra •Drug: recombinant human interferon gamma •Drug: placebo	NCT03332225

M1 macrophages play a key role in CKD. These macrophages increase plasma pro-inflammatory biomarkers such as TNF-α in patients, which may lead to chronic renal insufficiency. Furthermore, M2 macrophages are involved in chronic renal inflammation, especially in the repair phase, where M1 macrophages switch to M2 macrophages and secrete anti-inflammatory cytokines such as IL-10, IL-22, and TGF-β. M2 macrophages are involved more in the progression of fibrosis than M1 macrophages because they secret profibrotic factors such as TGF-β [100]. Adipocytes can play an important role in the management of macrophage polarization in adipose tissue. In healthy conditions, adipocytes promote M2-like polarization, but in obesity, adipocytes may favor the prevalence of M1-like macrophage polarization [101, 102]. Imbalance of inflammatory responses can cause many inflammatory insufficiencies, like what happens in IBD. In this case, lamina propria macrophages from patients with IBD have M1 phenotype rather than M2 phenotype, and as mentioned previously, these macrophages can produce a large amount of pro-inflammatory cytokines [103]. The polarization of macrophages is tied to glycolytic changes and oxidative phosphorylation (OXPHOS) metabolism. Indeed, M1 macrophages rely on glycolytic changes but M2 macrophages rely on fatty-acid-fueled OXPHOS. Many risk factors change macrophage polarization, including obesity and hypertension. These factors can lead to chronic and systemic inflammation by M1 macrophage activation. Obesity and hypertension can change target-organ damage by hormones, local inflammatory signals, and hypoxia-induced signaling or alteration in glycolytic- OXPHOS paradigm in macrophages [104]. Macrophage polarization processes and their regulation are very important for sufficient immune responses, and any skew in these processes may lead to some inflammation disorders [105].

## Exosomes involve in macrophage polarization

**Fig. 3:**
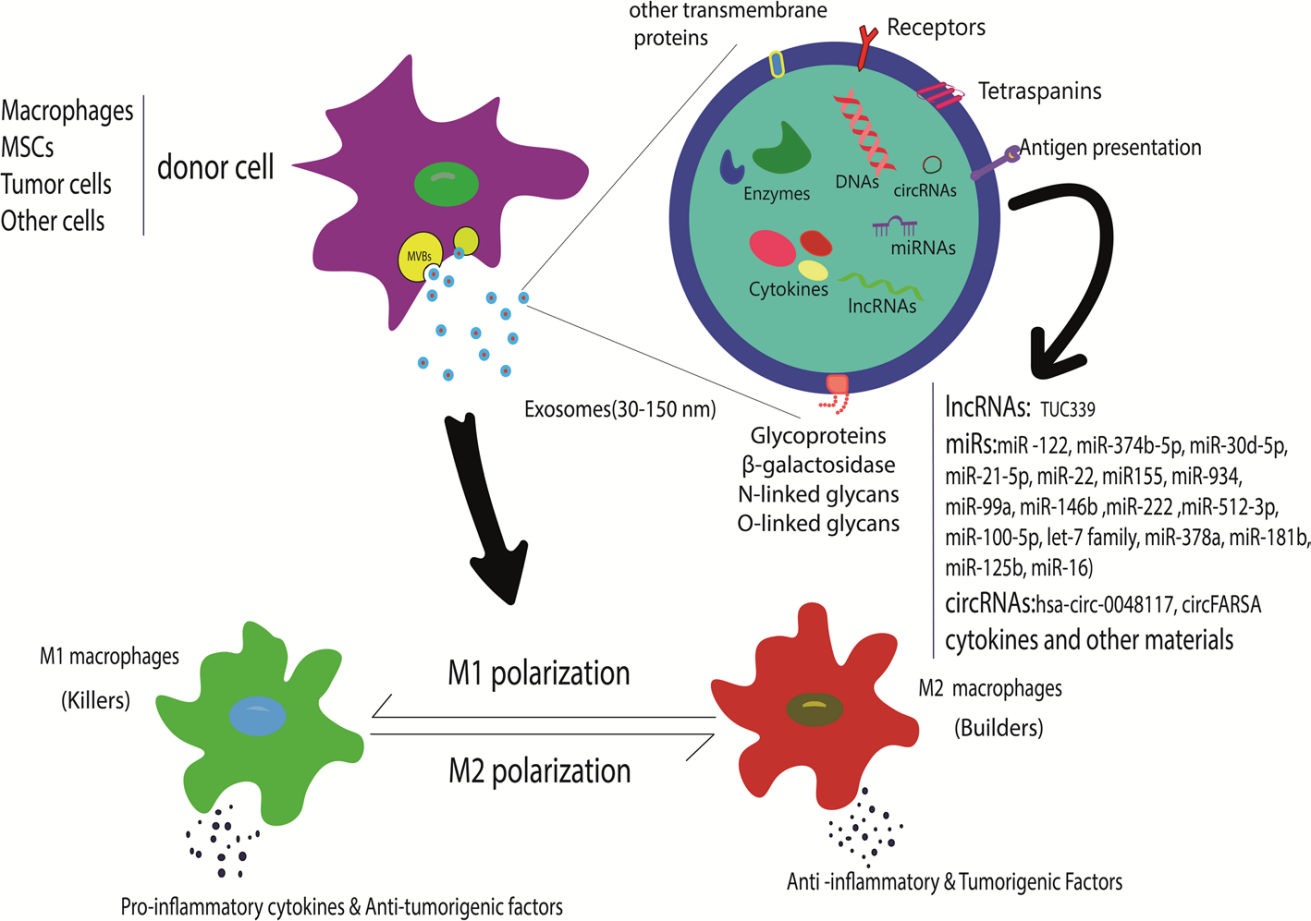
Exosomes are derived from different cells, including MSCs, tumor cells, or other cells that are present in the microenvironment such as macrophages. These exosomes can switch macrophage polarization according to their cargos. MSCs, mesenchymal stromal cells; lncRNA, long noncoding RNAs; miR, microRNAs; circRNA, circular RNA

Exosomes are a subset of nanoscale (30–150 nm) extracellular vesicles (EVs) that are released from almost every eukaryotic cell. Exosomes carry and deliver biological material, including proteins, lipids, saccharides, and genetic signals such as messenger ribonucleic acid (mRNAs), microRNAs (miRs), long noncoding RNAs (lncRNAs), deoxyribonucleic acid (DNAs), and circular RNAs (circRNAs). They have a very important role in cell communication. Exosomes have various effects on immune cell responses, stromal cells, and extracellular matrix (ECM), and can even alter them and their behaviors [106–108]. Exosomes derived from different cells play a key role in macrophage polarization processes. They are also able to change macrophage polarization. Recently, a research study demonstrated that M2-Exo causes a reprogramming of the M1 phenotype to the M2 phenotype (Fig. 3) [109]. Mesenchymal stromal cells (MSCs) have great potential to differentiate into many cell types. Numerous studies have demonstrated that exosomes secreted by MSCs have an important role in macrophage polarization [110, 111]. Exosomes present in serum have demonstrated involvement in IBD. A study reported that pregnancy zone protein (PZP) can be used as a biomarker in IBD [78].

Exosomes derived from MSCs of human bone marrow assist in the regulation of inflammatory responses. The systemic administration of these exosomes could substantially mitigate colitis in various models of IBD [112]. MSC exosomes have an important role in neuroinflammatory conditions. These exosomes can regulate macrophage polarization and change it toward an anti-inflammatory phenotype [113]. Also, MSC exosomes have a tumor-growth-suppressive effect by increasing inflammatory infiltration [111]. Pro-inflammatory bone-marrow-derived mesenchymal stem cells (BMMSCs) secrete exosomes that potentially promote macrophage M2 polarization. BMMSCs exosomes can reduce macrophage M1 polarization by regulation of the protein kinase B1/protein kinase B2 (AKT1/AKT2) signaling pathway and relieve myocardial injury [114, 115]. Fibronectin type III domain-containing protein 5 (FNDC5) is a transmembrane protein located in the cytoplasm that can increase BMMSC exosome secretion. This mechanism can promote M2 polarization by the nuclear factor-κB (NF-κB) signaling pathway [116, 117]. A study reported that administration of BMMSC-derived exosomes can induce macrophage M2 polarization, improve the inflammatory microenvironment, and promote fibrocartilage regeneration, especially at the tendon–bone interface [118].

Exosomes of adipose-derived stem cells (ADSCs) have an important role in obesity-associated inflammation and other metabolic disorders. They can induce anti-inflammation M2 macrophage polarization by carrying active STAT3 and inhibiting macrophage inflammatory responses [119]. ADSC exosomes have a critical role in myocardial repair after myocardial infection (MI). These exosomes can decrease lipopolysaccharides (LPS)-induced inflammation by activating sphingosine 1-phosphate (S1P), sphingosine kinase 1 (SphK1 or SK1), and sphingosine-1-phosphate receptor 1 (S1PR1) signaling, which leads to promotion of macrophage M2 polarization [120].

Exosomes derived from human umbilical cord mesenchymal stem cells (hUCMSCs) have involvement in regulation of macrophage polarization. They can inhibit M1 polarization and promote M2 polarization. These exosomes can inhibit the expression of tumor necrosis factor receptor-associated factor 1 (TRAF1), which has been shown to be involved in the macrophage M1 polarization mechanism and ameliorate steroid-resistant asthma (SSRA), an important clinical problem in asthma management [121]. Exosomes carry genetic signals such as miRs, which play a critical role in macrophage polarization. For example, mammary epithelial cells (MECs) can regulate immune system responses, secreting exosomes carrying exosomal miR-122. This miR can promote polarization of M1 macrophages by suppressing cytokine signaling 1 (SOCS1), STAT1, and STAT3 [122].

It has been demonstrated that exosomal miR-21-5p, originated from MSCs, can stimulate the polarization of M2 macrophages. It can reduce the inflammatory response and promote heart cell repair after myocardial ischemia–reperfusion injury [123]. It is reported that exosomal miR-21-5p released from MSCs has an important role in macrophage polarization. miR-21-5p induces M2 polarization by mediating phosphate and tensin homolog (PTEN) downregulation. This mechanism can support lung cancer cell growth and facilitate their invasion [110]. Upregulation of miR-374b-5p in exosomes, derived from hypoxic tubular epithelial cells (TECs), reduces SOCS1 expression and promotes M1 macrophage activation during renal ischemia–reperfusion injury (RIRI) conditions [124, 125]. Exosomes derived from polymorph nuclear neutrophils (PMNs) have an important role in sepsis-related ALI. These exosomes promote M1 macrophage activation by miR-30d-5p, which targets SOCS-1 and sirtuin 1 (SIRT1) in macrophages [126]. Atherosclerosis is an inflammatory disease that leads to clogging of blood vessels by the accumulation of lipids.

Exosomes, which originate from different cells, have a very important role in atherosclerosis. For example, exosomal miR-155 can enlarge inflammatory cytokines and M1 polarization markers such as cluster of differentiation 80 (CD80) and CD86, which are involved in the process of atherosclerosis through pro-inflammatory M1 polarization [127]. Some exosomal miRs such as miR-100-5p, miR-512-3p, let-7 family, and miR-21a-5p are derived from MSCs and have various properties. As these exosomal miRs can induce M2 macrophage polarization, they are capable of suppressing atherosclerosis [128, 129]. Bone-marrow-derived macrophages (BMDMs) can release exosomal miRs with anti-inflammatory properties, including miR-99a, miR-146b, and miR-378a, which are capable of promoting M2 polarization in BMDMs [127, 130]. Wu et al. generated exosomes with anti-inflammatory functions in atherosclerosis. These exosomes are derived from M2 macrophages and contain hexyl 5-aminolevulinate hydrochloride (HAL), which is FDA-approved and has an anti-inflammatory effect. This makes HALM2 exosomes a promising candidate for atherosclerosis therapy applying macrophage-derived exosomes [127, 131].

Exosomes can modulate immune responses in tumor cells. Tumor-derived exosomes (TEs) can change macrophage polarization, and could activate anti-inflammatory pro-tumorigenic M2 macrophage phenotypes or pro-inflammatory anti-tumorigenic M1 macrophage phenotypes, change the M1/M2 ratio in the TME, and promote tumor growth [132]. Melanoma-derived exosomes can upregulate specific macrophage polarization factors and promote mixed M1 and M2 macrophage phenotypes (128) Lung tumor cell-derived exosomes can reprogram macrophage metabolism and promote M2 macrophage polarization [133]. Exosomal miR-222 derived from adriamycin-resistant breast cancer cells can target phosphatases and PTEN gene, and activate the Akt pathway, so it can switch macrophage polarization to M2 phenotype and stimulate tumor growth [134, 135]. Exosomal miR-222 derived from adriamycin-resistant breast cancer cells can directly target phosphatase and the PTEN gene, activate the Akt pathway, convert macrophage polarization to M2 phenotype, and stimulate tumor growth by M2 macrophage polarization [136]. It has been reported that exosomes derived from hypoxic tumor cells can elevate oxidative phosphorylation in macrophages that originate from bone marrow by let-7a exosomal miR, which suppresses insulin-Akt-mammalian target of rapamycin (mTOR) signaling pathway and promotes M2-like macrophages [137].

Exosomal lncRNAs participate in macrophage polarization. For example, HCC-derived exosomes contain different levels of exosomal lncRNA TUC339 that regulate macrophage polarization [138]. It has been identified that circRNA, which is carried by exosomes, has important regulatory roles in different pathophysiological processes. For example, exosomal hsa-circ-0048117 has been upregulated in esophageal squamous cell carcinoma (ESCC). Upregulation of hsa-circ0048117 can promote M2 macrophage polarization and regulate ESCC progression. Another exosomal circRNA is circFARSA, which is highly expressed in tumor cells and is capable of inducing the promotion of M2 phenotypes and facilitating metastasis in non-small cell lung cancer (NSCLC) cells [139]. M1 macrophage-derived exosomes can be used as paclitaxel (PTX) nanocarriers and enhance antitumor activity of PTX [140]. These can also be tumor biomarkers such as exosomal miR-16, which are received by macrophages. These act as metastasis biomarkers in breast cancer [141], showing that exosomes can be employed for the treatment of cancer [59].

## Conclusion

Recently, it was confirmed that inflammation is a sign of chronic disease such as cancers, diabetes, cardiovascular, and neurologic system disorders. This review points out that the plasticity features of macrophages give them a critical role in inflammatory conditions. Macrophage polarization can be switched from pro-inflammatory with anti-tumorigenic macrophages (M1-like) phenotype to anti-inflammatory with pro-tumorigenic (M2-like) phenotype. In addition. The balance between different types of macrophages plays a key role in immune system dysfunctions. Exosomes as vehicles in cell communication have serious involvement in macrophage polarization. Cancerous cells can modulate immune cell responses by their secreted exosomes. There are many immune-based strategies that have been established for cancer therapy such as cancer vaccines. Various agents have been used for delivering of medicines.

Exosomes can change macrophage polarization and promote or prevent different subtypes of macrophage population via their cargos, such as miRs, cricRNAs, and lncRNAs. Numerous studies have reported that these nano-sized vesicles could be engineered for medical aims and developed as delivery systems for immune system modulation. However, there are several unsolved problems in the clinical application of exosomes as biologically derived nanovesicles. Examples of this are selecting the appropriate exosome isolation method, ensuring purity of exosomes, and identifying an efficient method for exosome modification. In conclusion, further studies on macrophage polarization mechanisms and its related pathways are needed to elucidate exosomes’ roles in these pathways and their therapeutic potential in the development of immunotherapies for various medical aims.

## Data Availability

Not applicable.

## Abbreviations

MPS: Mononuclear phagocyte system
DCs: Dendritic cells
CNS: Central nervous system
TAM: Tumor-associated macrophages
CSF-1: Colony-stimulating factor-1
M-CSF: Macrophage-colony-stimulating factor
CSF-1R: Colony-stimulating factor-1 receptor
Th1: T-helper lymphocytes type 1
TNF-α: Tumor necrotic factor-α
MCP-1: Monocyte chemo attractant protein-1
IL: Interleukin
IFNs: Interferons
iNOS: Inducible nitric oxide synthase
CXCLs: C–X–C motif chemokine ligands
CCR: C–C chemokine receptor
CXCR: C–X–C motif chemokine receptor
YM1: Chitinase-like protein 3
MgI1: Macrophage and granulocyte inducer-form 1
DNMT: DNA methyl transferase
DNAm: DNA methylation
RA: Rheumatoid arthritis
NAFLD: Non-alcoholic fatty liver disease
TZDs: Thiazolidinediones
PPAR-γ: Peroxisome proliferator-activated receptor gamma
T2D: Type 2 diabetes
TDAG8: T-cell death-associated gene 8
GNP: Gold nanoparticle
ALI: Acute lung injury
ARDS: Acute respiratory distress syndrome
AS-IV: Astragaloside IV
AMPK: Adenosine monophosphate-activated protein kinase
HCC: Hepatocellular carcinoma
SLE: Systemic lupus erythematosus
IBD: Inflammatory bowel diseases
CF: Cystic fibrosis
TME: Tumor microenvironment
STATs: Signal transducer and activator of transcription
CMs: Cardiac macrophages
TGF-β: Transforming growth factor-β
CKD: Chronic kidney disease
OXPHOS: Oxidative phosphorylation
EVs: Extracellular vesicles
DNA: Deoxyribonucleic acid
miRs: MicroRNAs
lncRNA: Long noncoding RNAs
circRNAs: Circular RNAs
mRNA: Messenger ribonucleic acid
ECM: Extracellular matrix
MSCs: Mesenchymal stromal cells
BMMSCs: Bone marrow-derived mesenchymal stem cells
AKT: Protein kinase B
FNDC5: Fibronectin type III domain-containing protein 5
NF-κB: Nuclear factor-κB
ADSCs: Adipose derived stem cells
MI: Myocardial infection
LPS: Lipopolysaccharides
S1P: Sphingosine 1-phosphate
SI: Sterile inflammation
SphK1 or SK1: Sphingosine kinase 1
S1PR1: Sphingosine-1-phosphate receptor 1
hUCMSCs: Human umbilical cord mesenchymal stem cells
TRAF1: Tumor necrosis factor receptor-associated factor 1
SSRA: Steroid-resistant asthma
MECs: Mammary epithelial cells
SOCS1: Suppressor of cytokine signaling 1
PMNs: Polymorph nuclear neutrophils
PTEN: Phosphate and tensin homolog
PZP: Pregnancy zone protein
TECs: Tubular epithelial cells
RIRI: Renal ischemia–reperfusion injury
SIRT1: Sirtuin 1
BMDMs: Bone-marrow-derived macrophages
CD: Cluster of differentiation
HAL: Hexyl 5-aminolevulinate hydrochloride
TEs: Tumor-derived exosomes
mTOR: Mammalian target of rapamycin
ESCC: Esophageal squamous cell carcinoma
NSCLC: Non-small cell lung cancer
PTX: Paclitaxel
CCL: Chemokine (C–C motif) ligand
TLR: Toll-like receptor
VEGF: Vascular endothelial growth factor
ICAM: Intercellular adhesion molecule
DC-SIGN: Dendritic cell-specific ICAM-grabbing non integrin
IRF5: Interferon regulatory factor 5
HDAC3: Histone deacetylase 3
MerTK: Myeloid epithelial reproductive tyrosine kinase
NOS: Nitric oxide synthase
NO: Nitric oxide
ODC: Ornithine decarboxylase
SRM: Spermidine synthase
SMOX: Spermine oxidase
siRNA: Small interfering RNA
DAMP: Danger-associated molecular patterns
PAMP: Pathogen-associated molecular patterns
M2-Exo: M2-derived exosomes
FDA: US Food and Drug Administration
